# Treatment Landscape of Nonmetastatic Castration-Resistant Prostate Cancer: A Window of Opportunity

**DOI:** 10.3390/jpm11111190

**Published:** 2021-11-12

**Authors:** Fernando López-Campos, Antonio Conde-Moreno, Marta Barrado Los Arcos, Antonio Gómez-Caamaño, Raquel García-Gómez, Asunción Hervás Morón

**Affiliations:** 1 Deparment Radiation Oncology, Hospital Universitario Ramón y Cajal, 28034 Madrid, Spain; 2 Deparment Radiation Oncology, Hospital Universitario y Politécnico La Fe, 46010 Valencia, Spain; conde_ant@gva.es; 3 Deparment Radiation Oncology, Hospital Universitario de Navarra, 31008 Pamplona, Spain; marta.barrado.losarcos@navarra.es; 4 Deparment Radiation Oncology, Hospital Clínico Universitario de Santiago de Compostela, 15706 Santiago de Compostela, Spain; antonio.gomez.caamano@sergas.es; 5 Deparment Radiation Oncology, Hospital Clínico Universitario de Valencia, 46010 Valencia, Spain; raquelitagarcia@gmail.com

**Keywords:** nonmetastatic castration-resistant prostate cancer, second generation anti-androgens, apalutamide, enzalutamide, darolutamide, overall survival

## Abstract

The treatment for nonmetastatic castration-resistant prostate cancer (nmCRPC) is a highly unmet medical need. The classic treatment approach for these patients—androgen deprivation therapy (ADT) alone—until metastatic progression is now considered suboptimal. Several randomized phase III clinical trials have demonstrated significant clinical benefits—including significantly better overall survival (OS)—for treatments that combine ADT with apalutamide, enzalutamide, and darolutamide. As a result, these approaches are now included in treatment guidelines and are considered a standard of care. In the present article, we discuss the changing landscape of the management of patients with nmCRPC.

## 1. Introduction

Prostate cancer (PC) is the most common genitourinary tumor in men worldwide, being associated with a significant epidemiological burden with more than 1.4 million cases worldwide and more than 375,000 associated deaths [1]. Most of these tumors are diagnosed in the early stages, allowing for a curative treatment with radical intention. However, some patients are diagnosed with “de novo” metastatic disease and others (10–20%) develop metastasis in the first five years of follow-up after receiving curative treatment [2]. In these patients, androgen deprivation therapy (ADT) has been the mainstay of treatment for many years, either with LHRH analogues (leuprorelin, goserelin, and/or triptorelin) or exceptionally with LHRH antagonists [3]. However, the recent emergence in the market of oral LHRH antagonists like relugolix may change this scenario in the near future [4]. In summary, at the present time, we have complementary treatments that allow us to both improve overall survival and maintain the quality of life of these patients [5,6,7,8]. However, the development of castration-resistant prostate cancer (CRPC), which represents a lethal stage of the disease, occurs in practically all advanced cases and after a variable time from the onset of ADT. The median survival in castration-resistant cancer patients is around 60 months in different studies [9,10,11] and is significantly lower in patients presenting with metastatic disease [12,13].

Castration resistance is defined as the progression of the disease despite testosterone levels being in the castration range [14]. Progression is understood to be the presence of at least one of the following circumstances:

- Biochemical progression: three consecutive increases in PSA, 1 week apart, resulting in two increases of 50% over PSA nadir, with PSA > 2 ng/mL.
- Radiological progression: appearance of new lesions, either two or more new bone lesions in the bone scan, or a new soft tissue lesion assessable using RECIST 1.1 criteria.

In a significant percentage of patients in whom ADT is started due to local or biochemical relapse after local treatment, castration resistance is observed without evidence of metastasis by conventional techniques (CT and/or bone scintigraphy). The estimated prevalence of CRPC is close to 18% of patients with PC [2] and nmCRPC is estimated to represent approximately 30% of CRPC cases [15].

The mechanisms driving the progression of androgen dependent PC to CRPC are unclear. Continuous androgen receptor signaling, despite circulating androgen depletion and androgen receptor blockade, is postulated as one of the key events for the development of CRPC. For this reason, therapies directed against the androgen pathway may remain effective, even though the disease is known to be castration resistant. In this sense, the excellent survival results of the studies with second-generation hormonal treatments (apalutamide, enzalutamide, and darolutamide) [9,10,11] have led to a change in the therapeutic strategy. The mechanism of action of these treatments is similar and is based on the selective inhibition of androgen receptors through direct binding to the receptor’s ligand binding domain, thus preventing its nuclear translocation, its binding to DNA, and transcription mediated by the receptor. These drugs lack agonist activity on androgen receptors and have powerful antitumor activity, reducing the proliferation of tumor cells and increasing their apoptosis. Its use has been approved by the main regulatory agencies such as the Food and Drug Administration (FDA) and the European Medicines Agency (EMA) for the treatment of high-risk nmCRPC, understood as nmCRPC with PSA doubling time (PSADT) ≤ 10 months [16,17], and its indication having been reflected in the main clinical practice guidelines on genitourinary tumors [18,19,20].

## 2. Pivotal Clinical Trials

The results obtained in the SPARTAN, PROSPER, and ARAMIS studies, whose main objective was metastasis-free survival (MFS) [9,10,11], are considered essential for the indication of second-generation hormonal treatments in high-risk nmCRPC.

Brave et al. [21] showed similarities between these studies, such as phase III, double-blind trials with 2:1 randomization of drug versus placebo; patients with PSADT ≤ 10 months (subgroup <6 months and ≥6 months); the allowed inclusion of pelvic adenopathies of up to 2 cm in the SPARTAN and ARAMIS studies; the authorized inclusion of patients who had or were receiving treatment with bone protective agents; conventional imaging techniques that were performed at baseline and every 16 weeks (thoraco-abdomino-pelvic CT or MRI and bone scintigraphy) to determine the absence of metastasis and disease progression, based on the response evaluation criteria in solid tumors (RECIST), version 1.1 [22]; and the main endpoints were MFS, overall survival (OS), and time to initiation of cytotoxic therapy.

Regarding the main differences in design, the SPARTAN study randomized the presence of locoregional lymph node disease to N0 and N1; there was ignorance of the PSA figures for the ARAMIS patients; and pelvic adenopathic progression was considered a metastatic event in MFS in the PROSPER study. While the PROSPER study analyzed time to PSA progression as a secondary endpoint, the SPARTAN study studied time to metastasis, progression-free survival, and time to symptomatic progression, and the ARAMIS study examined the time to pain progression and time to the onset of the first skeletal event (Table 1).

Regarding the characteristics of the patients included, the median age in the three studies was 74 years. In the SPARTAN study, the median PSADT was 4.4 (apalutamide) and 4.5 (placebo) months. The study showed that 71.5% of the patients treated with apalutamide and 70.8% of the placebo group had a PSADT <6 months. Furthermore, 16.5% of patients treated with the drug presented lymph node disease compared to 16.2% of the placebo group. It also showed that 77.3% of the apalutamide group presented ECOG 0 compared to 77.8% of the placebo group, and 89.8% of these did not take bone protective agents compared to 90.3% of the placebo group. In the PROSPER study, the median PSADT was 3.8 (enzalutamide) and 3.6 months (placebo). In this study, 77% of the patients included in both groups belonged to the PSADT subgroup <6 months. Moreover, 80% of patients with enzalutamide presented ECOG 0 compared to 82% of the placebo group, and 89% of patients with the drug did not take bone protective agents compared to 90% of the placebo group. In the ARAMIS study, the median PSADT was 4.4 (darolutamide) and 4.7 months (placebo). For the patients treated with darolutamide, 70% belonged to the PSADT subgroup <6 months, compared to 67% in the placebo group. Furthermore, 17% of patients treated with the drug had adenopathic disease compared to 29% of the placebo group. The study also revealed that 68% of the darolutamide group had ECOG 0 versus 71% of the placebo group, and 97% of the patients in the exploratory arm were not taking bone resorption inhibitors versus 94% of the placebo group (Table 2). The three trials included patients who had in the majority of the cases received treatment for their primary tumor, while the percentage of patients included without treatment for their primary tumor was variable (41% in the ARAMIS, 28% in the PROSPER, and 23% in the SPARTAN study).

In the initial analysis, after a median follow-up of 20.3 months in SPARTAN, 18.5 months in PROSPER, and 17.9 months in ARAMIS, MFS increased significantly in patients who received apalutamide (HR: 0.28; 95% CI: 0.23–0.35), enzalutamide (HR: 0.29; 95% CI: 0.24–0.35), and darolutamide (HR: 0.42; 95% CI: 0.35–0.50) compared to the placebo groups [23]. In a subsequently published direct random-effects meta-analysis (I^2^ = 79%) [24], MFS was in favor of drug-treated patients compared to the placebo-treated patients (HR: 0.32; 95% CI: 0.25–0.41). In the indirect comparison, the MFS was in favor of apalutamide (HR: 0.73; 95% CI: 0.55–0.97) and enzalutamide (HR: 0.71; 95% CI: 0.54–0.93) compared to darolutamide, without differences in the indirect comparison between apalutamide and enzalutamide (HR: 1.03; 95% CI: 0.78–1.73). None of the studies provided data for significant differences in overall survival in the preliminary results [25]. It was in the final analysis of each of these trials, where apalutamide, after a median follow-up of 52 months, decreased the risk of death in the intention-to-treat population by 22%, with a median of 73.9 vs. 59.9 months (HR: 0.78; 95% CI: 0.64–0.96; *p* = 0.016) [10]. After a median follow-up of 48 months, enzalutamide was associated with a significant reduction in the risk of death of 27% compared to the placebo, with a median of 67 vs. 56.3 months (HR: 0.73; 95% CI, 0.61–0.89; *p* = 0.001) [11], and after a median follow-up of 29 months, the risk of death was 31% lower in the darolutamide group than in the placebo group, without reaching the median overall survival (HR: 0.69; 95% CI: 0.53–0.88; *p* = 0.003) [9].

Regarding the rest of the secondary objectives analyzed (Table 3), in the PROSPER study biochemical progression-free survival was 37.2 vs. 3.9 months (HR: 0.07; 95% CI: 0.05–0.08) and time to subsequent antineoplastic treatment was 39.6 vs. 17.7 months (HR: 0.21; 95% CI: 0.17–0.26). The incidence of adverse effects (AEs) adjusted to treatment time was similar to that reported in the initial publication, with grade ≥III adverse effects in 48% of patients treated with enzalutamide compared to 27% of patients in the control group, with falls, asthenia, and high blood pressure the most common AEs. In terms of quality of life (QoL), there was an initial non-significant decrease in the treatment group followed by a later improvement, with benefits in pain progression, symptoms, and functional status [26]. In the SPARTAN study, progression-free survival was 40.5 vs. 14.7 months (HR: 0.29; 95% CI: 0.24–0.36); time to symptomatic progression had an HR of 0.45 and a 95% CI of 0.32–0.63; and time to subsequent antineoplastic treatment had an HR of 0.44 and a 95% CI of 0.29–0.66. Regarding QoL, a significant improvement was observed from the first year in the treatment group [27]. Among the AEs, it is worth highlighting an increase in rashes (23% vs. 5.5%), hypothyroidism (8.1% vs. 2%), and fractures (11.7% vs. 6.5%) without exceeding grade ≥III in 6% of each group, with asthenia and hypertension being the most common AEs in both arms [10]. Finally, in the ARAMIS study, the time to pain progression was 40.3 vs. 25.4 months (HR: 0.65; 95% CI: 0.53–0.79); bone event had an HR of 0.48 and a 95% CI of 0.29–0.82; and time to subsequent antineoplastic treatment had an HR of 0.33 and a 95% CI of 0.23–0.47. In QoL, the scores of the scales used were similar in both groups and slightly better than those in the treatment group [28].

Previous pooled meta-analyses, performed with immature data from interim analysis with median survival not reached, have demonstrated that darolutamide is the treatment option with the most favorable profile of side effects and the least drug interactions [24,32,33]. With this in mind, we must consider the differences in the design of the studies that would preclude carrying out representative direct comparisons of the true safety profiles of these drugs in this clinical setting.

## 3. Discussion

Currently, we do not have evidence in terms of efficacy or toxicity to determine what is superior in the treatment of first line high risk nmCRPC, leaving the choice of agent to the preferences of the physician and the patient. However, several comparative analyses have tried to discern differences in the toxicity profiles of these treatments, as well as the benefit of selecting a specific drug in order to consider a possible treatment sequence with second-generation hormonal treatments.

No prospective randomized trials have been conducted to evaluate which sequencing is appropriate for patients progressing from nmCRPC to mCRPC. For patients who progressed in the pivotal studies, the therapeutic options used were abiraterone plus prednisone, enzalutamide, darolutamide (if not previously employed), and docetaxel or cabazitaxel. To a smaller percentage, radium-223, sipuleucel-T, bicalutamide, carboplatin, cisplatin, cyclophosphamide, dexamethasone, ethinylestradiol, flutamide, investigational antineoplastic drugs (PD-L1-inhibitor plus apalutamide, pTVG-HP plasmid DNA-vaccine), and rucaparib were used [9,10,11].

In both the ARAMIS and PROSPER trials, most of the patients who progressed in the experimental group underwent treatment with docetaxel [9,11]. The percentages of patients who received subsequent treatment were lower than those reported in the SPARTAN trial. Enzalutamide has shown that it can be used in the context of nmCRPC and mCRPC, and the PREVAIL study [12] supported its use in mCRPC before chemotherapy, so it could be planned as a logical sequence also in this context, taking into account that its sequencing with another second-generation hormonal treatment is still unclear. The concept of second progression-free survival (PFS2) emerged in this area, initially defined as the time from the start of first-line treatment in high-risk nmCRPC to progression to first treatment for mCRPC. It was incorporated as an exploratory objective in the SPARTAN trial and the benefit for the evolution of a patient of an early introduction of a second-generation hormonal treatment for high-risk nmCRPC before progression to mCRPC was emphasized [34]. In the SPARTAN study, patients had access to abiraterone + prednisone within the study after initial progression, leading to the consideration of apalutamide followed by abiraterone + prednisone as a possible sequence of second-generation hormonal treatments in these patients. Although these conclusions are highly debated, this exploratory objective has not been developed with enzalutamide and darolutamide. Apalutamide extended median PFS2 by 14.4 months versus the placebo and reduced the hazard of second progression or death by 45% versus the placebo (HR: 0.55; 95% CI, 0.46–0.66; *p* < 0.0001. However, the PFS2 results were reported in conjunction with the apalutamide and placebo groups and were not stratified by the first subsequent therapy received [29]. Further research is needed to draw more robust conclusions for the sequencing treatment of these patients.

Another important aspect in the evaluation of these pivotal trials was the crossover of patients from the placebo to the experimental arm after blinding rupture (18.6% PROSPER, 19% SPARTAN, and 31% ARAMIS) [9,10,11]. On the other hand, several comparative safety studies have been carried out using various methodologies such as network meta-analyses and matching-adjusted indirect comparison [24,25,32,33,35]. Altavilla et al. evaluated the comparative safety of apalutamide, enzalutamide, and darolutamide in nmCRPC through a network meta-analysis (NMA) using summary data from SPARTAN, PROSPER, and ARAMIS [36]. NMAs do not adjust for differences in trial populations in contrast to MAICs (matching-adjusted indirect comparisons). In that NMA, darolutamide exhibited a lower risk of falls and mental deterioration than apalutamide, and reported a lower risk of falls, fatigue (in all grades), hypertension, and mental deterioration compared to enzalutamide. These results were consistent with the study results when risk differences were used. The MAICs carried out, such as that of Halabi et al., confirm these data, which points to their validity [37]. However, despite these studies, it is also noteworthy that the monitoring and toxicity records have been more exhaustive in SPARTAN than in PROSPER and ARAMIS (4 vs. 16 weeks), and both SPARTAN and PROSPER currently have a higher follow-up. Another important aspect is the variation in the risks of AEs in the placebo arms and the lack of inferential statistical methodology in these studies. We must not forget that patients with nmCRPC are mostly asymptomatic patients; thus, avoiding toxicity and drug interactions at this stage is essential to maintain their quality of life.

## 4. Special Situations and Drug Interactions

It is of vital importance to identify and differentiate the unalike clinical situations that we may encounter in our healthcare practice based on the baseline characteristics of the patients. Although we know that the chronological age does not always correspond to the real age of the patient, the tolerance in the elderly may differ from that of younger patients, with different metabolisms. In the same manner, it is important to take into account those patients with significant comorbidity or those who are polymedicated, in which certain nuances must be taken into account when prescribing these treatments [38,39,40].

### 4.1. Liver Insufficiency

No dose adjustment of enzalutamide is necessary in patients with mild, moderate, or severe hepatic impairment (Child–Pugh class A, B, or C, respectively). However, an increased half-life of enzalutamide has been observed in patients with severe hepatic impairment. There is also no need to adjust the dose of apalutamide and darolutamide in patients with previous mild or moderate hepatic impairment. Nevertheless, in severe cases, its use is not recommended since there are no data on this patient population and apalutamide is eliminated mainly via the liver.

### 4.2. Renal Insufficiency

No dose adjustment is necessary for these treatments in patients with mild or moderate renal impairment. Caution is recommended in patients with severe renal failure or end-stage renal disease, since these drugs have not been studied in this patient population. If treatment is initiated in this clinical situation, adverse reactions should be monitored, and the starting dose reduced.

### 4.3. Drug Interactions

Apalutamide and enzalutamide are powerful enzyme inducers and increase the synthesis of many enzymes and metabolic transporters in a way that can interact with a wide range of commonly used drugs that are substrates for these enzymes or transporters. This would determine the decrease in their plasma concentrations and a decrease in their effectiveness. Therefore, when treatment with these drugs is started, a review of concomitant medication should be performed.

#### 4.3.1. Effect of Exposure on Other Drugs

- **Drug metabolizing enzymes:** Apalutamide is a strong inducer of CYP3A4 and CYP2C19, and a weak inducer of CYP2C9, while enzalutamide is a strong inducer of CYP3A4 and a moderate inducer of CYP2C9 and CYP2C19. Darolutamide is a mild inducer of CYP3A4. Concomitant use of apalutamide/enzalutamide with medicinal products metabolized by CYP3A4, CYP2C19, and CYP2C9, or medicinal products that are substrates of UGT (glucuronic conjugating enzyme) may reduce the exposure of these drugs. Co-administration of apalutamide and enzalutamide with warfarin and coumarin-type anticoagulants should be avoided. In the event that such administration is necessary, additional controls of the International Normalized Ratio (INR) should be performed (Table 4).
- **Drug transporters:** Apalutamide is a weak inducer of the gp protein (P-gp), the breast cancer resistance protein (BCRP), and the organic anion transporter polypeptide 1B1 (OATP1B1). Therefore, concomitant use of drugs that are substrates for these proteins can reduce exposure to them. In vitro data indicate that enzalutamide may be an inhibitor of P-gp and the inhibition of BCRP and MRP2 cannot be ruled out. Theoretically, induction of these transporters is also possible, and their net effect is currently unknown. Darolutamide is an inhibitor of BCRP and OATP 1B1 and 1B3, so co-administration with certain drugs can increase the plasma concentrations of these substrates.
- **Medications that prolong the QT interval:** Because androgen deprivation therapy can prolong the QT interval, concomitant use with substances that prolong the QT interval or that may induce Torsade de Pointes (class IA or III antiarrhythmic drugs and antipsychotics) should be carefully evaluated.

#### 4.3.2. Effect of Other Drugs on Exposure

- **Medicines that inhibit CYP2C8 and CYP3A4:** CYP2C8 and CYP3A4 play a role in the elimination of apalutamide and enzalutamide and in the formation of their active metabolites. No initial dose adjustment is necessary when apalutamide is co-administered with a strong CYP2C8 inhibitor or with a strong CYP3A4 inhibitor. During treatment with enzalutamide, it is recommended to avoid the use of strong CYP2C8 inhibitors, although no dose adjustment is necessary with concomitant CYP3A4 inhibitors. The use of darolutamide with a combination of a P-gp inhibitor and a strong CYP3A4 inhibitor increases drug exposure, thereby increasing the risk of adverse reactions (Table 5).
- **Medicines that induce CYP3A4, CYP2C8, or P-gp:** Inducers of CYP3A4 or CYP2C8 have no clinically relevant effects on the pharmacokinetics of apalutamide and enzalutamide. The use of strong CYP3A4 and P-gp inducers with darolutamide may decrease their plasma concentration and is not recommended.

## 5. Future Directions

The frequent use of new generation imaging techniques in the different clinical scenarios of PC has raised the suitability of their application in high-risk nmCRPC [41]. However, using these type of imaging techniques requires changing clinical practice according to their results, without currently having evidence to do so. We have data that indicate if we use the same selection criteria used in the pivotal studies of high-risk nmCRPC and perform a PET/PSMA in these patients, up to 55% of them would be identified as having metastatic disease [42]. The RADAR III recommendations indicate that we only have to consider PET/PSMA if the PSADT is less than 6 months, as long as we are considering the indication of an available treatment for mCRPC (abiraterone, enzalutamide, or docetaxel). However, the indication for these treatments was given by conventional imaging techniques (CT and bone scan), which are used in both the COU-302 [13] and PREVAIL [12] studies. In conclusion, there is no consensus in this section, and in fact, there was no consensus in the latest APCCC consensus publication on whether these type of imaging techniques should be conducted in these patients [43]. However, it was performed in mid-2019, and although we did have the results of the primary objectives of the SPARTAN, PROSPER, and ARAMIS studies, we did not have the mature data at that time of global survival that would surely have changed the voting.

Nevertheless, stage migration and treatment management changes as a result of the use of new generation imaging techniques (PET/PSMA, PET/choline, or whole-body MRI), do not necessarily improve clinical outcomes. Improvement in each clinical subgroup separately does not necessarily have an associated improvement in the prognosis of the group as a whole (Will Rogers effect) [44]. Further evidence is needed to change clinical practice based on next-generation imaging techniques in the management of advanced prostate cancer. Various publications address how clinical trials should be designed incorporating these advances in imaging techniques in order to evaluate the true value of them [45].

Although the standard treatment for these patients at the moment is second-generation hormonal treatment plus conventional ADT [20], from the conceptual point of view it makes sense to carry out advanced imaging techniques in order to perform stereotactic body radiation therapy (SBRT) on identified metastatic lesions, since SBRT could eliminate resistant cell clones to conventional ADT and systemic treatment. This treatment approach could demonstrate increased overall survival, by keeping the rest of the disease under control, and is currently being studied in certain clinical trials (NCT03503344, NCT02685397). Another approach that is being evaluated within trials (NCT02816983, NCT02192788), supported by positive data from retrospective studies [46], would be the possibility of performing SBRT in patients with a limited number of metastatic lesions, identified by new generation imaging techniques, maintaining the conventional ADT in order to delay the start of new systemic treatments, with the consequent benefit for the patient. In the coming years, we will have more data on the integration of new imaging techniques and new treatment modalities that will allow us to assess whether optimizing the treatment of our patients is possible (Table 6).

## 6. Conclusions

nmCRPC is a heterogeneous disease that, in high-risk patients, can have an aggressive and lethal course. Treatments directed against the androgen pathway may continue to be effective in this clinical setting. In this sense, the excellent results in survival and quality of life of the studies with second-generation hormonal treatments (apalutamide, enzalutamide, and darolutamide), associated with their excellent safety profile, have led to a change in the management of these patients, and they have been transferred to the clinical practice guidelines and approved by the main regulatory agencies. Some aspects, such as the integration of new generation imaging techniques in the identification of patients belonging to this clinical scenario, are still to be defined, as well as the application of metastasis-directed therapies that allow for optimization of the results published to date.

## Figures and Tables

**Table 1:** Design characteristics of the pivotal trials in high-risk nmCRPC.

	SPARTAN	PROSPER	ARAMIS
**Inclusion Criteria**	Absence of metastases (CT scan/bone scintigraphy) Baseline PSA ≥ 2 ng/mL. PSA progression. PSADT ≤ 10.		
	N1 allowed	Only N0	N1 allowed
**Stratification Factors**	PSADT ≥ 6 m or PSADT < 6 m Use of bone targeting agents		
	Presence of nodal metastases	-	-
**Primary Endpoints**	Metastasis-free survival		
**Key Secondary Endpoints**	Time to PSA progression, quality of life, **overall survival**		
	T to symp prog, T to SSE, PFS, T to chemo, PFS2	T to pain prog, PFS	T to pain prog, T to SSE
**Follow-up**	Imaging: every 16 weeks		
	Toxicity: every 4 weeks (C1-6), 8 weeks (7–13), 16 weeks (after C13)	Toxicity: every 16 weeks	Toxicity: every 16 weeks
**Pt/Physician blinded to PSA**	Yes	Yes	No

**Table 2:** Baseline patients’ characteristics in in the pivotal studies carried out in high-risk nmCRPC.

	SPARTAN A vs. P *n* = 806 *n* = 401	PROSPER E vs. P *n* = 933 *n* = 468	ARAMIS D vs. P *n* = 955 *n* = 554
**Age median** (yr)	74 vs. 74	74 vs. 73	74 vs. 74
**PSADT median** (mo) **PSADT <6 mo** (%)	4.4 vs. 4.5 71.5 vs. 70.8	3.8 vs. 3.6 77 vs. 77	4.4 vs. 4.7 70 vs. 67
**Nodal disease** (%)	16.5 vs. 16.2	-	17 vs. 29
**Performance status 0** (%)	77.3 vs. 77.8	80 vs. 82	68 vs. 71
**Bone targeted therapy** (%)	10.2 vs. 9.7	11 vs. 10	3 vs. 6

E: Enzalutamide, P: placebo, A: apalutamide, D: darolutamide, yr: years, mo: months, PSADT: prostate specific antigen duplication time, %: percentage.

**Table 3:** Obtained results in the pivotal studies carried out in high-risk nmCRPC.

	SPARTAN [10,29] (*n* = 1207) A vs. P	PROSPER [11,30] (*n* = 1401) E vs. P	ARAMIS [9,31] (*n* = 1509) D vs. P
**Metastases free survival (MFS)** (mo) Primary objective	40.5 vs. 16.2 **HR 0.28** 95% CI (0.23–0.35) *p* < 0.001	36.6 vs. 14.7 **HR 0.29** 95% CI (0.24–0.35) *p* < 0.001	40.4 vs. 18.4 **HR 0.41** 95% CI (0.34–0.50) *p* < 0.001
**Overall survival (OS)** (mo) Secondary objective	73.9 vs. 59.9 **HR 0.78** 95% CI (0.64–0.96) *p* = 0.016	67 vs. 56.3 **HR 0.73** 95% CI (0.61–0.89) *p* = 0.001	Alive 3 years 83% vs. 77% **HR 0.69** 95% CI (0.53–0.88) *p* = 0.003
**Time to beginning of QT** (mo) Secondary objective	NR vs. NR **HR 0.63** 95% CI (0.49–0.81) *p* = 0.0002	-	No QT in 3 years 83% vs. 75% **HR 0.58** 95% CI (0.44–0.76) *p* < 0.001
**PSA progression free survival** (mo) Secondary objective in PROSPER Exploratory objective in SPARTAN and ARAMIS	40.5 vs. 3.7 **HR 0.07** 95% CI (0.06–0.09) *p* < 0.0001	37.2 vs. 3.9 **HR 0.07** 95% CI (0.05–0.08) *p* < 0.001	33.2 vs. 7.3 **HR 0.13** 95% CI (0.11–0.16) *p* < 0.001
**Time to 1st skeletal event** Secondary objective	-	-	No EE in 3 years 96% vs. 92% **HR 0.48** 95% CI (0.29–0.82 *p* = 0.005
**Time to pain progression** (mo) Secondary objective	-	-	40.3 vs. 25.4 **HR 0.65** 95% CI (0.53–0.79) *p* < 0.001
**Time to next antineoplastic therapy** (mo) Secondary objective in PROSPER Exploratory objective in ARAMIS	-	66.7 vs. 19.1 **HR 0.29** 95% CI (0.25–0.35) *p* < 0.001	NR vs. NR **HR 0.33** 95% CI (0.23–0.47) *p* < 0.001
**Second progression free survival** Exploratory objective	55.6 vs. 41.2 **HR 0.55** 95% CI (0.46–0.66) *p* < 0.0001	-	-
**Time to symptomatic progression** Secondary objective	NR vs. NR **HR 0.57** 95% CI (0.44–0.73) *p* < 0.0001	-	-
**Progression free survival** (mo) Secondary objective in SPARTAN Exploratory objective in ARAMIS	40.5 vs. 14.7 **HR 0.29** 95% CI (0.24–0.36) *p* < 0.001	-	36.8 vs. 14.8 **HR 0.38** 95% CI (0.32–0.45) *p* < 0.001

E: Enzalutamide, P: placebo, A: apalutamide, D: darolutamide, MFS: metastasis free survival, mo: months, HR: hazard ratio, CI: confidence interval, p: *p*-value, PSA: prostate specific antigen, NR: not reached, OS: overall survival, QT: chemotherapy, EE: skeletal-related events.

**Table 4:** Effect of second-generation hormonal treatments on other drugs.

Substrates	Apalutamide	Enzalutamide	Darolutamide
CYP3A4 (fentanyl, oxycodone, rivaroxavan, amlodipine, sinvastatin, tamsulosin, solifenacin, alprazolam)	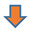	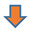	_
CYP2C9 (phenytoin, warfarin, acenocoumarol, celecoxib, losartan, fluvastatin)	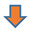	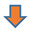	_
CYP2C19 (omeprazole, lansoprazole, propanolol, diazepam)	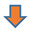	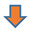	_
UGT (levothyroxine, valproic acid)	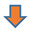	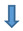	_
P-gp (colchicine, dabigatran, etexilate, digoxin)	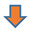	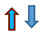	_
BCRP (furosemide, fluvastatin, atorvastatin, rosuvastatin)	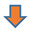	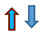	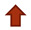
OATP1B1 (lapatinib, methotrexate, repaglinide)	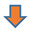	NO	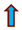

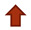
 Strong effect with augmented exposition to the drug (high risk of toxicity); 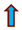 Weak effect with augmented exposition to the drug (low risk of toxicity); 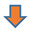 Strong effect with decreased exposure to the drug (high risk of loss of efficacy); 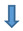 Weak effect with decreased exposure to the drug (low risk of efficacy loss).

**Table 5:** Effect of other drugs on second-generation hormonal treatments.

Inhibitors/Inducers	Apalutamide	Enzalutamide	Darolutamide
CYP3A4-inhibitor (ketoconazole, ritonavir, clarithromycin)	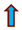	_	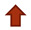
CYP32C8-inhibitor (gemfibrozil, clopidogrel)	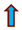	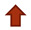	_
CYP3A4-inductor (rifampicin)	_	_	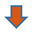
P-gp-inductor (rifampicin, phenobarbital, phenytoin)	_	_	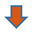

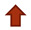
 Strong effect with increased exposure to ARI (high risk of toxicity); 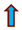 Weak effect with increased exposition to ARI (low risk of toxicity); 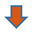 Strong effect with decreased exposition to ARI (high risk of loss of efficacy); ARI: Androgen receptor inhibitor.

**Table 6:** Ongoing clinical trials with second-generation hormonal treatments in oligometastatic CRPC and nmCRPC.

Study	Official Name	Type	State	Ending Stipulated Date
NCT 04108208	A Multicenter, Randomized, Double-Blind, Placebo-Controlled, Phase IV Study of Apalutamide in Chinese Subjects with Non-Metastatic Castration-Resistant Prostate Cancer (NM-CRPC)	Phase IV	Recruiting	April 2027
NCT03800784	Study of 18F-DCFPyL PET/CT, for Detection of Radiological Progression in Patients with Metastatic (M+) and Non-metastatic (M0) Castration Resistant Prostate Cancer Receiving Standard Androgen Receptor Targeted Treatment	Phase II–III	Recruiting	January 2024
NCT02685397	Management of Castration-Resistant Prostate Cancer with Oligometastases (PCS IX)	Phase II–III	Recruiting	April 2025
NCT04070209	The Role of Therapeutic Layering of Stereotactic Body Radiotherapy on Darolutamide in the Management of Oligoprogressive Castration Resistant Prostate Cancer: A Pilot Phase II Trial	Phase II	Recruiting	November 2027
NCT03503344	Apalutamide With or Without Stereotactic Body Radiation Therapy in Treating Participants with Castration-Resistant Prostate Cancer (PILLAR)	Phase II	Recruiting	December 2024
NCT04319783	Darolutamide + Consolidation Radiotherapy in Advanced Prostate Cancer Detected by PSMA	Phase II	Recruiting pending	June 2026
NCT 03569280	A First-in-Human Study to Determine the Safety, Pharmacokinetics and Efficacy of KPG-121 When Administered with Enzalutamide, Abiraterone, or Apalutamide in Subjects with Non-Metastatic or Metastatic Castration-Resistant Prostate Cancer	Phase I	Recruiting	December 2021
NCT04122976	Darolutamide Observational Study in Non-metastatic Castration-resistant Prostate Cancer Patients	Observational	Recruiting	April 2025
NCT04567875	Evaluation of Cardiotoxicity and Hypertension in Patients with Non Metastatic Castration Resistant Prostatic Carcinoma	Observational	Recruiting	September 2022
NCT02588001	Japanese Research for Patients with Non-metastatic Castration Resistant Prostate Cancer-Enzalutamide	Observational	Active Recruiting ended	September 2021

## Data Availability

Not applicable.
